# ACE2 Expression in Organotypic Human Airway Epithelial Cultures and Airway Biopsies

**DOI:** 10.3389/fphar.2022.813087

**Published:** 2022-03-11

**Authors:** Qianyu Chen, Shenna Langenbach, Meina Li, Yuxiu C. Xia, Xumei Gao, Matthew J. Gartner, Elizabeth A. Pharo, Sinéad M. Williams, Shawn Todd, Nadeene Clarke, Sarath Ranganathan, Michelle L. Baker, Kanta Subbarao, Alastair G. Stewart

**Affiliations:** ^1^ Department of Biochemistry and Pharmacology, School of Biomedical Science, University of Melbourne, Parkville, VIC, Australia; ^2^ ARC Centre for Personalized Therapeutics Technologies, University of Melbourne, Parkville, VIC, Australia; ^3^ Department of Microbiology and Immunology, University of Melbourne, Parkville, VIC, Australia; ^4^ CSIRO, Health and Biosecurity Business Unit, Australian Centre for Disease Preparedness, Geelong, VIC, Australia; ^5^ Murdoch Children’s Research Institute, The Royal Children’s Hospital, Parkville, VIC, Australia; ^6^ Department of Pediatrics, Melbourne Medical School, University of Melbourne, Parkville, VIC, Australia; ^7^ WHO Collaborating Centre for Reference and Research on Influenza at The Peter Doherty Institute for Infection and Immunity, Melbourne, VIC, Australia

**Keywords:** COVID-19, SARS-CoV-2, asthma, ALI, organoids, cytokine, interferon, steroid

## Abstract

Coronavirus disease 2019 (COVID-19) caused by infection with the severe acute respiratory syndrome coronavirus 2 (SARS-CoV-2) is an acute respiratory disease with systemic complications. Therapeutic strategies for COVID-19, including repurposing (partially) developed drugs are urgently needed, regardless of the increasingly successful vaccination outcomes. We characterized two-dimensional (2D) and three-dimensional models (3D) to establish a physiologically relevant airway epithelial model with potential for investigating SARS-CoV-2 therapeutics. Human airway basal epithelial cells maintained in submerged 2D culture were used at low passage to retain the capacity to differentiate into ciliated, club, and goblet cells in both air-liquid interface culture (ALI) and airway organoid cultures, which were then analyzed for cell phenotype makers. Airway biopsies from non-asthmatic and asthmatic donors enabled comparative evaluation of the level and distribution of immunoreactive angiotensin-converting enzyme 2 (ACE2). ACE2 and transmembrane serine proteinase 2 (TMPRSS2) mRNA were expressed in ALI and airway organoids at levels similar to those of native (i.e., non-cultured) human bronchial epithelial cells, whereas furin expression was more faithfully represented in ALI. ACE2 was mainly localized to ciliated and basal epithelial cells in human airway biopsies, ALI, and airway organoids. Cystic fibrosis appeared to have no influence on ACE2 gene expression. Neither asthma nor smoking status had consistent marked influence on the expression or distribution of ACE2 in airway biopsies. SARS-CoV-2 infection of ALI cultures did not increase the levels of selected cytokines. Organotypic, and particularly ALI airway cultures are useful and practical tools for investigation of SARS-CoV-2 infection and evaluating the clinical potential of therapeutics for COVID-19.

## Introduction

Coronavirus disease 2019 (COVID-19) is caused by severe acute respiratory syndrome coronavirus 2 (SARS-CoV-2). The symptoms of COVID-19 when present are highly varied, but may include fever, dry cough, shortness of breath and loss of taste and sense of smell (Agyeman et al., 2020; Zhang et al., 2020). Severe cases of COVID-19 can lead to hypoxia, respiratory failure, and death. Dexamethasone improves survival of hospitalized patients (Group et al., 2021). Extensive numbers of clinical trials have been carried on a wide range of agents. Molnupiravir and paxlovid have shown significant reduction in risk of hospital admission or death (Mahase, 2021a; b). Both agents have been authorized by FDA as emergency use for the treatment of mild-to-moderate COVID-19. The burden of infection in many countries is reduced but not eliminated by vaccination. Control of COVID-19 is complicated by the emergence of variant viruses that are more transmissible (e.g., the delta variant, the omicron variant) or against which current vaccines show reduced effectiveness (beta variant). Vaccine hesitancy, an inevitable fraction of the vaccinated population who fail to mount an adequate immune response and the potential development of new virus strains resistant to vaccines further emphasize the necessity to develop additional treatment approaches for COVID-19, including repurposed drugs and novel agents.

Case studies of hospitalized COVID-19 patients suggest older age, hypertension, diabetes, cardiovascular disease, and higher number of comorbidities are associated with disease severity (Li et al., 2020; Wu and McGoogan, 2020; Zhang et al., 2020). Surprisingly, asthma, chronic obstructive pulmonary disease (COPD), and allergic disease have not been identified as risk factors for COVID-19 severity (Chhiba et al., 2020; Li et al., 2020; Lovinsky-Desir et al., 2020; Zhang et al., 2020). This lack of susceptibility of patients with lung diseases contrasts with infections by rhinovirus (RV), respiratory syncytial virus (RSV), or influenza virus, that exacerbate the inflamed airways in these prevalent airway/lung diseases (Tan et al., 2020). Patients with cystic fibrosis (CF), a condition characterized by infection by bacterial and viral pathogens (De Boeck and Amaral, 2016), were also underrepresented in a COVID-19 cohort (Colombo et al., 2020). Interestingly, a recent study using individually linked community and SARS-CoV-2 test data has found that severe COVID-19 symptoms are associated with use of maintenance medication for asthma (an index of disease severity) (Bloom et al., 2021). Meta-analysis of COVID-19 clinical studies indicated that COPD is associated with increased odds of poor clinical outcomes in patients with COVID-19 (Gerayeli et al., 2021).

A range of airway cellular models have been developed for investigation of respiratory infections. Freshly isolated and uncultured primary cells provide the closest model to the native condition, but are limited in utility by a short *ex vivo* lifespan, access to donors, and cell numbers obtained. The A549 type II alveolar epithelial carcinoma cell line and BEAS-2B simian virus 40 (SV40)-transformed bronchial epithelial cell lines have been commonly used in respiratory research (Keenan et al., 2014; Hillyer et al., 2018). However, these cell lines lack the *in vivo* architecture and phenotypic diversity, and their transformed proliferative pathways may distort cellular pharmacology. Most human epithelial cell lines are not susceptible to SARS-CoV-2 infection *in vitro*, except for CaCo2 and Calu3 cells (Chu et al., 2020). More (patho)physiologically relevant cellular models are therefore needed for COVID-19.

The air-liquid interface (ALI) culture is a routine approach to differentiate epithelial cells (Chen and Schoen, 2019) for viral infection studies, including influenza (Wu et al., 2016; Pharo et al., 2020), RSV (Xia et al., 2017), coronaviruses (Dijkman et al., 2013), and SARS-CoV-2 (Zhu et al., 2020; Marsh et al., 2021). Recently, self-renewing organoid models have rapidly advanced understanding of stem cell biology, organogenesis, and human pathologies (Dutta et al., 2017). The use of organoids to study influenza virus (Zhou et al., 2018) and adeno-associated virus (Meyer-Berg et al., 2020) may bridge the gaps between *in vitro* and *in vivo* infection models. In recent work, liver organoids (Yang et al., 2020) and lung bud tip organoids (Lamers et al., 2020) both showed susceptibility to SARS-CoV-2 infection.

We have systematically compared airway cell lines, ALI, and airway organoid culture to freshly isolated human bronchial epithelial cells to benchmark the expression and distribution of angiotensin converting enzyme 2 (ACE2), the primary receptor for SARS-CoV-2. Biopsies from a cohort of asthmatic and non-asthmatic subjects (Keenan et al., 2018; Li et al., 2019) were also evaluated for the distribution and abundance of ACE2 to provide a further benchmark for comparison with airway epithelial cultures. Cytokine levels and morphogenic changes in ALI cultures after SARS-CoV-2 infection confirmed their utility in investigations of COVID-19.

## Methods

### Primary Human Bronchial Epithelial Cell Brushings

Twelve pediatric cystic fibrosis (CF) patients and four non-CF participants at the Royal Children’s Hospital Melbourne were enrolled and consented following a clinical management indication for flexible bronchoscopy. The clinical characteristics of the subjects with CF are shown in Supplementary Table S1. Ethical approval was obtained from Royal Children’s Hospital Melbourne (HREC 25054) and the University of Melbourne (HREC 2056658).

The bronchial epithelial brushing cells obtained during flexible bronchoscopy using cytology brushes (Olympus) were dislodged by agitation, then seeded onto collagen-coated 25 cm^2^ cell culture flasks in Bronchial Epithelial Growth Medium (BEGM, Lonza) supplemented with Single Quots (Lonza) and Amphotericin B (Life Technologies) at 37°C in air containing 5% CO_2_. The native cells were sedimented onto cytocentrifuge slides (Cytospin 2, Shandon, 350 rpm, 10 min) left to dry overnight and fixed in pre-chilled methanol for 5 min and then washed with PBS, prior to storage or staining.

### Primary Human Bronchial Epithelial Cell

Primary human bronchial epithelial cells (HBEC) were established as previously described (Schuliga et al., 2009) from airways tissue obtained under approval from University of Melbourne (HREC 1750014) and Alfred Hospital (336/13). Briefly, the bronchial epithelial cells were obtained by scraping the inner surface of airway with a no. 23 scalpel blade. The cells were resuspended in BEGM medium and seeded on collagen-coated 25 cm^2^ cell culture flasks. Cells were cultured at 37°C in a humidified atmosphere containing 5% CO_2_.

### Cell Lines

Well-characterized immortalized BCi cell line (Walters et al., 2013) were cultured in BEGM as HBEC cells. African green monkey kidney epithelial cells, Vero cell line (ATCC Cat#CCL-81) were cultured at 37°C, 5% CO_2_ in Minimum Essential Media (MEM) (Media Preparation Unit, Peter Doherty Institute) supplemented with 5% Fetal Bovine Serum (FBS) (Sigma-Aldrich), 1X penicillin/streptomycin (Gibco), 1X GlutaMAX (Gibco), and 15 mM HEPES (Gibco). Vero/hSLAM cells (European Collection of Authenticated Cell Cultures [ECACC] #04091501) were grown at 37°C, 5% CO_2_ in MEM with 7% FBS (Sigma-Aldrich), 1X penicillin/streptomycin (Gibco), 1X GlutaMAX (Gibco), and 15 mM HEPES (Gibco) and 0.4 mg/ml geneticin (Gibco).

### Air-Liquid Interface

ALI cultures were generated as previously described (Prodanovic et al., 2017; Xia et al., 2017; Pharo et al., 2020). Briefly, cells used for ALI culture were originally passaged in submerged culture in BEGM prior to medium change to PneumaCult™ Ex Plus medium (STEMCELL Technologies), supplemented with hydrocortisone (STEMCELL Technologies). Upon reaching 80% confluency, the cells were dissociated using an animal component-free cell dissociation kit (STEMCELL Technologies), then seeded on fibrillar collagen (0.03 mg/ml rat tail collagen)-coated 24-well Corning Transwell^®^ (surface area: 0.33 cm^2^) with 0.4 µm pore polyester membrane inserts (Corning). The cells were cultured in PneumaCult™ Ex Plus for 4 days until 100% confluency was reached. Upon confluency, the cells were “air-lifted” by removing the growth medium from the apical surface and replacing the basal medium with PneumaCult™ ALI Medium (STEMCELL Technologies), supplemented with hydrocortisone and heparin (STEMCELL Technologies) according to manufacturer’s instructions. The PneumaCult™ ALI medium was changed every second day and apical surfaces were washed with PBS (no Ca^+^, no Mg^2+^, Gibco) every week, from 1 week after air lifting. One day before viral infection or drug treatment, if not otherwise specified, the hydrocortisone concentration in the medium was reduced to 100 nM according to the previous protocol (Prodanovic et al., 2017; Xia et al., 2017). Bright-field microscopy detectable cilia beating was captured using Olympus IX53 microscope equipped with QImaging optiMOS high speed camera (100 frames/s). The cells were fixed in 10% neutral buffered formalin (NBF, Grale Scientific) for 10 min. The membrane was excised from the transwell and cut into 4 pieces (Levardon et al., 2018) for whole-mount staining or embedded in paraffin and sectioned for staining.

### Airway Organoids

Airway organoids were generated by a protocol modified (Broutier et al., 2016; Sachs et al., 2018) as follows. The elastomeric stencil silicone mask was used to standardize the size and shape of the Matrigel droplet (Supplementary Figure S1A). The diameter of the mask can be adjusted to fit different culture plates or bio-printing settings. Briefly, an elastomeric stencil silicone mask was placed in the 48-well plate (diameter: 11 mm). The outer diameter and inner diameter of the silicone mask are 8 and 5 mm. The inner surface bounded by the mask was then coated with 25 µL of 1% BSA (Sigma) for 1 h at 37°C. The residue was removed and washed once with PBS. The plate was left to dry and pre-warmed in the incubator. The NHBE cells cultured in BEGM were dissociated as described above and resuspended in 50% growth factor reduced (GFR) Matrigel^®^ (Corning) at a density of 3,000 cells/well. A 25 µL droplet was added onto the inner surface of the silicone mask. After solidification of the droplet, 250 µL of the airway organoid medium (Supplementary Table S2) was added into the plate. The medium was changed every second day. All the organoids were cultured for 3 weeks. The development of airway organoids was recorded using the same microscope, camera and methodology as for ALI. The organoids were fixed in 10% NBF for 30 min as described previously (Broutier et al., 2016; Dekkers et al., 2019). All the tips and tubes in contact with organoids were coated with 1% BSA to minimize cell adherence.

### SARS-CoV-2 Preparation

SARS-CoV-2 human isolate (BetaCoV/Australia/VIC01/2020), provided by the Victorian Infectious Diseases Reference Laboratory (VIDRL) was passaged in Vero hSLAM cells and stored at −80°C. Virus stocks were quantified by virus titration as the median tissue culture infectious dose (TCID_50_) in Vero CCL-81 cells as previously described (Mills et al., 2021). All work with infectious virus was performed inside a biosafety II cabinet, in a biosafety containment level 3 facility, and personnel wore powered air-purifying respirators (3M TR-315A VERSAFLO) or P2 masks.

### SARS-CoV-2 Infection

SARS-CoV-2 infection experiments were conducted at two sites: Department of Microbiology and Immunology, University of Melbourne; Australian Centre for Disease Preparedness (ACDP), CSIRO. At University of Melbourne, primary normal human bronchial epithelial cell (NHBE)-ALI and BCi-ALI cultures were inoculated at a multiplicity of infection (MOI) of 0.1 and 1 (10^4^ and 10^5^ TCID_50_) of SARS-CoV-2 via the apical surface for 1 h at 37°C. The inoculum was removed, and the apical surface washed twice with PBS (second wash is the D0 sample). Up to day six, daily apical samples were obtained by PBS washing for 30 min at 37°C. Infectious virus titer was quantified as TCID_50_/ml as previously described (Mills et al., 2021). At ACDP, NHBE ALI cultures were inoculated with SARS-CoV-2 at a MOI of 0.05 for 1 h at 37°C (Marsh et al., 2021). The inoculum was removed, and the apical surface washed with ALI medium. Cells were cultured at 37°C for 48 h. Cells were lysed for RT-qPCR analysis. Basolateral supernatants were collected and gamma-irradiated with a minimum of 50 kGy while on dry ice (Steritech, Dandenong) prior to cytokine analyses. All work with infectious virus was performed in a biosafety containment level 3 or 4 facility.

### Rhinovirus Infection and Poly I:C Treatment

To compare the impact of SARS-CoV-2 infection with other respiratory infections, rhinovirus (RV) and double-stranded RNA synthetic mimetic Poly I:C were used. RV16 strain (VR-283, ATCC) was prepared as previously described (Xia et al., 2017). NHBE ALI cultures were inoculated with rhinovirus at a MOI of 1 for 1 h at 37°C. The inoculum was removed, and the apical surface washed with PBS. Cells were cultured at 37°C for 48 h. NHBE ALI cultures treated with 10 μg/ml Poly I:C were cultured at 37°C for 24 h. Basolateral supernatants were collected for cytokine analysis.

### Immunofluorescence Staining

To perform immunofluorescence staining of cell type markers (acetylated tubulin for ciliated cell, MUC5AC for goblet cell, CC10 for club cell, and KRT5 for basal cell), ACE2, and SARS-CoV-2, samples were blocked in 5% goat serum (Sigma)/0.1% Triton X-100 in PBS for 1 h at room temperature. The samples were incubated with primary antibodies (Supplementary Table S3) diluted in 1% BSA/0.1% Triton X-100 in PBS overnight at 4°C, followed by incubation of secondary antibodies (Supplementary Table S3) for 1 h at room temperature. Nuclei and actin filaments were stained with DAPI (Santa Cruz) and Alexa Fluor^®^ 488 Phalloidin (Cell Signaling). DAKO fluorescent mounting medium (DAKO) was used for mounting. Airway organoids were stained, cleared, and mounted as described previously (Dekkers et al., 2019). Specifically, Poly (2-hydroxyethyl methacrylate) (Poly-HEMA, Sigma) coated plates were used for immunofluorescence staining. For ALI samples, all the staining steps were carried out in Eppendorf tubes. The membrane piece was transferred into the mounting media dropped onto a microscope slide bordered by double-sided sticky tape (Scotch 3M) to avoid disruption of the 3D organoid structure. The confocal images were acquired using a Zeiss LSM880 Airyscan Fast confocal microscope (Biological Optical Microscopy Platform, University of Melbourne) and analyzed using Imaris 9.2 and FIJI ImageJ software.

### Immunohistochemical Staining of ACE2

The airway biopsies used in this study were obtained from subjects recruited from the Melbourne Epidemiological Study of Childhood Asthma (MESCA) cohort (all aged 42 years at the time of biopsy) (Ward et al., 2008; Qiao et al., 2017; Li et al., 2019). Asthma severity in this study was classified by using contemporary Global initiative for Asthma guidelines as described in detail in (Ward et al., 2008). The demographic data of the donors are provided in Supplementary Table S4. Paraffin-embedded sections of human airways and ALI cultures differentiated from NHBE cells were stained with ACE2 (Abcam) using three-layer immunoperoxidase protocol. ACE2 expression level was determined by a semi-quantitative IHC scoring method. In parallel with ACE2 staining, selected sections were stained with anti-α-smooth muscle actin (Dako, 1:400) as positive control and isotype IgG as negative control. The ACE2 staining was qualitatively scored from 0 to 5 by an experienced operator and confirmed by an additional operator (each blinded to group allocations), with 0 denoting no staining and 5 indicating heavy uniform and extensive staining (Supplementary Figure S4).

### Isolation of Total RNA and RT-qPCR

RNA was extracted to measure the expression of cell type marker genes (ciliated cell: DNAH1, DNAH5, FOXJ1, and TEKTIN; secretory cell: MUC5AC, MUC5B, and TFF3; basal cell: ITGA6 and KRT5), SARS-CoV-2 receptors, and SARS-CoV-2. Total RNA from SARS-CoV-2 infected cells was extracted using MagMAX™-96 Total RNA Isolation Kit (Invitrogen) or TRIzol (Invitrogen). Total RNA from all the other experiments was extracted using illustra RNAspin Mini RNA Isolation Kit (GE Healthcare). RNA extracts were reverse transcribed using a High-Capacity RNA-to-cDNA Kit (Applied Biosystem). Real-time PCR was performed on QuantStudio 6 Flex Real-Time PCR System using iTaq^®^ Universal SYBR^®^ green supermix (Bio-Rad). 18S ribosomal RNA was used as reference. Primer sequences are documented in Supplementary Table S5.

### Quantification of Cytokine Levels in Supernatant

Supernatants from SARS-CoV-2 experiments were collected for measurement of the chemokine IL-8 (BD) and the cytokines IL-6 (BD) and GM-CSF (BD) by ELISA, following the manufacturer’s instructions. High throughput cytokine/chemokine measurement was done by using Bio-Plex Pro™ Human Cytokine Grp I Panel 27-Plex (Bio-Rad), following the manufacturer’s instruction.

### Statistical Analysis

All data were statistically analyzed by GraphPad Prism 7.0 (GraphPad, San Diego, CA, United States) and presented as the mean ± standard error of mean (SEM) for n individual experiments in cell lines, for n individual donors of primary epithelial cell culture or median ± interquartile range for n individual donors of MESCA samples. For analysis of one independent variable, one-way analysis of variance (ANOVA) with the Dunnett’s *post-hoc* test, or non-parametric Kruskal-Wallis test, and non-parametric Mann-Whitney test were used. Two-way ANOVA with Bonferroni *post-hoc* tests were used for analysis in experiments with two independent variables. *p* < 0.05 was considered to be statistically significant.

## Results

### Air-liquid interface and bronchial organoids can recapitulate *in vivo* bronchial epithelial structures

**FIGURE 1:**
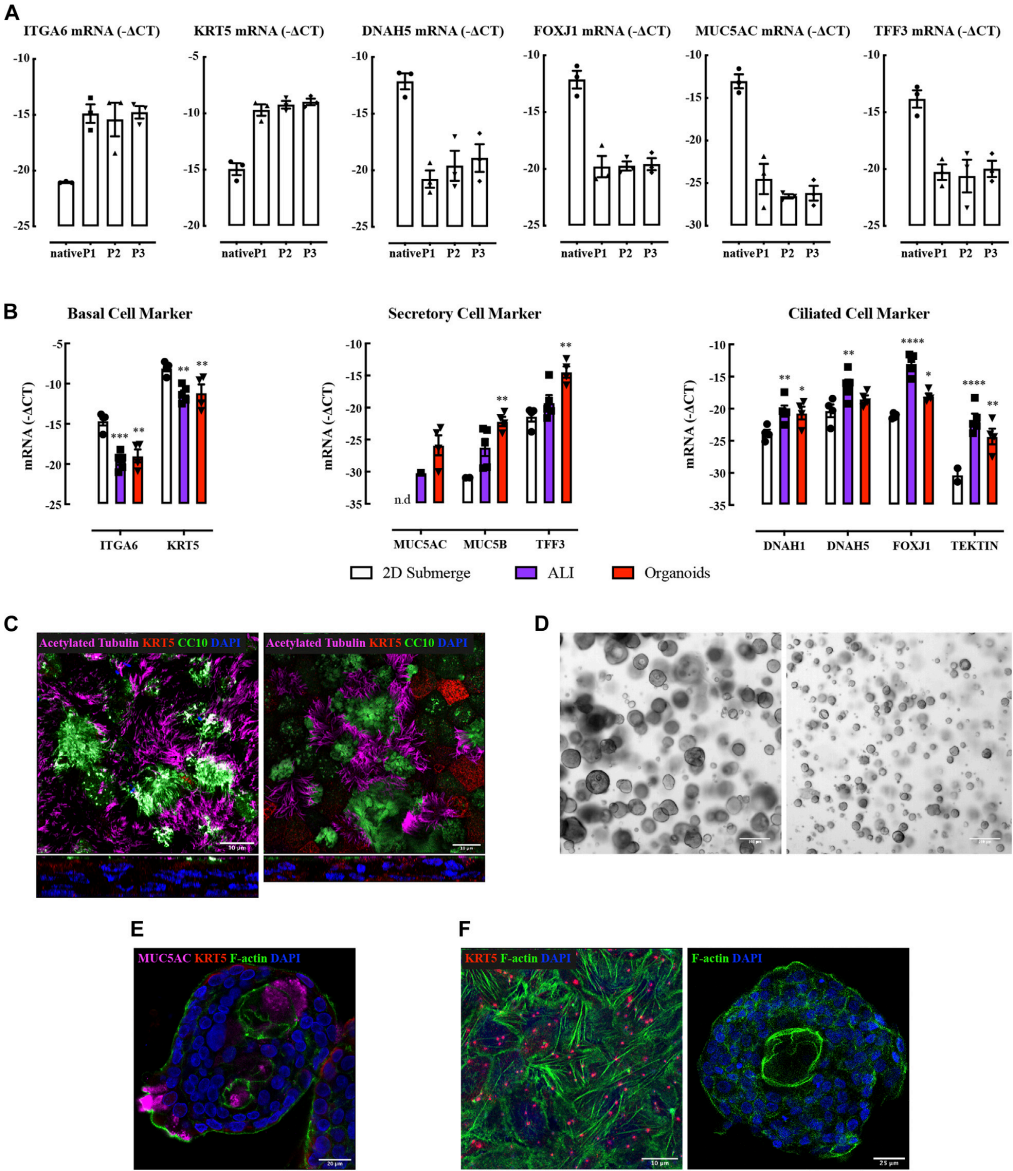
Characterization of human bronchial epithelial brushings, air-liquid interface culture, and bronchial-derived organoids. **(A)** Quantitative real-time PCR (qRT-PCR) analysis of ciliated (DNAH5, FOXJ1), secretory (MUC5AC, TFF3), and basal cell (ITGA6, KRT5) genes. Gene expression is expressed as −∆CT (Log2). Data are presented as mean and SEM for n = 3 independent cultures analyzed without culture (native) or at stated passage numbers (P1-3). **(B)** Gene expression levels of cell type markers in submerged NHBE cells, ALI, and organoids. Gene expression is expressed as −∆CT (Log_2_). Data are presented as mean and SEM for *n* = 4 independent NHBE and organoids, *n* = 5 for ALI. **(C)** Immunofluorescence staining of acetylated tubulin (magenta), CC10 (green), KRT5 (red), and nucleus (blue) in NHBE (left) and BCi (right) cell derived ALI. Confocal fluorescence images are presented en face (top) and from vertical sections (bottom). **(D)** Bright-field images of bronchial organoids at Day 20 (two independent NHBE cell cultures, 4x objective). **(E)** Immunofluorescence staining of MUC5AC (magenta), F-actin (green), KRT5 (red), and nucleus (blue) in NHBE cell derived bronchial organoid. **(F)** Immunofluorescence staining of F-actin (green), KRT5 (red), and nucleus (blue) in NHBE cell derived ALI (left) and organoid (right) cultures.

Bronchial brushings of ciliated cells, goblet cells, club cells, and basal cells were fixed prior to *ex vivo* culture and cell types identified by cell-specific markers: acetylated tubulin, MUC5AC, CC10, p63, and KRT5 respectively (Supplementary Figure S1B). Native epithelial cells were cultured to generate submerged primary cultures which showed increased basal cell marker gene expression and decreased ciliated and secretory cell marker gene expression, consistent with ciliated and secretory cells failing to propagate in conventional two-dimensional culture (Figure 1A). To assess persistence of ciliated and secretory cells *in vitro*, native bronchial epithelial cells were embedded in Matrigel^®^. Cell clusters generated spheroid-like structures within 48 h (Supplementary Video S1) and beating cilia were observed for 14 days (Supplementary Video S2). However, these cells did not grow or proliferate.

We used normal human bronchial epithelial cells (NHBE) or immortalized human airway basal epithelial cell line BCi cells (Walters et al., 2013) to generate ALI cultures. Each cell preparation differentiated into ciliated and secretory cells under ALI culture conditions (Figures 1B,C), consistent with previous observations (Keenan et al., 2014; Prodanovic et al., 2017; Xia et al., 2017).

Advanced DMEM/F12 supplemented with FGF7, FGF10, R-Spondin 1, Noggin, A83-01, Y-27632, and SB202190 supported the differentiation of NHBE, but not BCi cells, into bronchial organoids that showed inter and intra donor size variation (Figure 1D). Moreover, a lumen was not consistently obtained. Beating cilia oriented toward both the outer surface of the organoid and the lumen were identified after 2 weeks of culture (Supplementary Videos S3, S4; Supplementary Figure S1C). These “bipolar” organoid structures have rarely been reported (Zhou et al., 2018), whereas “unipolar” organoid structures are commonly reported (Danahay et al., 2015; Sachs et al., 2019). Mucin secretion was also bipolar (Figure 1E).

The expression of genetic markers of ciliated cells was higher in ALI whereas secretory cell marker expression was higher in organoids (Figure 1B). F-actin was evident as abundant filaments throughout cells at bottom layer in ALI culture; more limited amounts of F-actin appeared to be restricted to the cell membrane in organoids (Figure 1F). Mechanical influences on organotypic culture differentiation require further investigation.

### ACE2 and TMPRSS2 are Restored by Air-Liquid Interface Culture and Bronchial Organoids Culture

To evaluate the utility of airway epithelial *in vitro* models in viral infection studies, the expression of toll-like receptor (TLR) genes was assessed. The TLRs were divided into four groups based on the expression pattern among native epithelial cells, submerged NHBE cells, submerged BEAS-2B cells, ALI, and organoids (Supplementary Figure S2A). In general, the TLR expression level in submerged cell cultures was lower than that in native cells (non-cultured). Culture in ALI and organoid format elevated TLR expression to levels similar to those measured in native cells.

**FIGURE 2:**
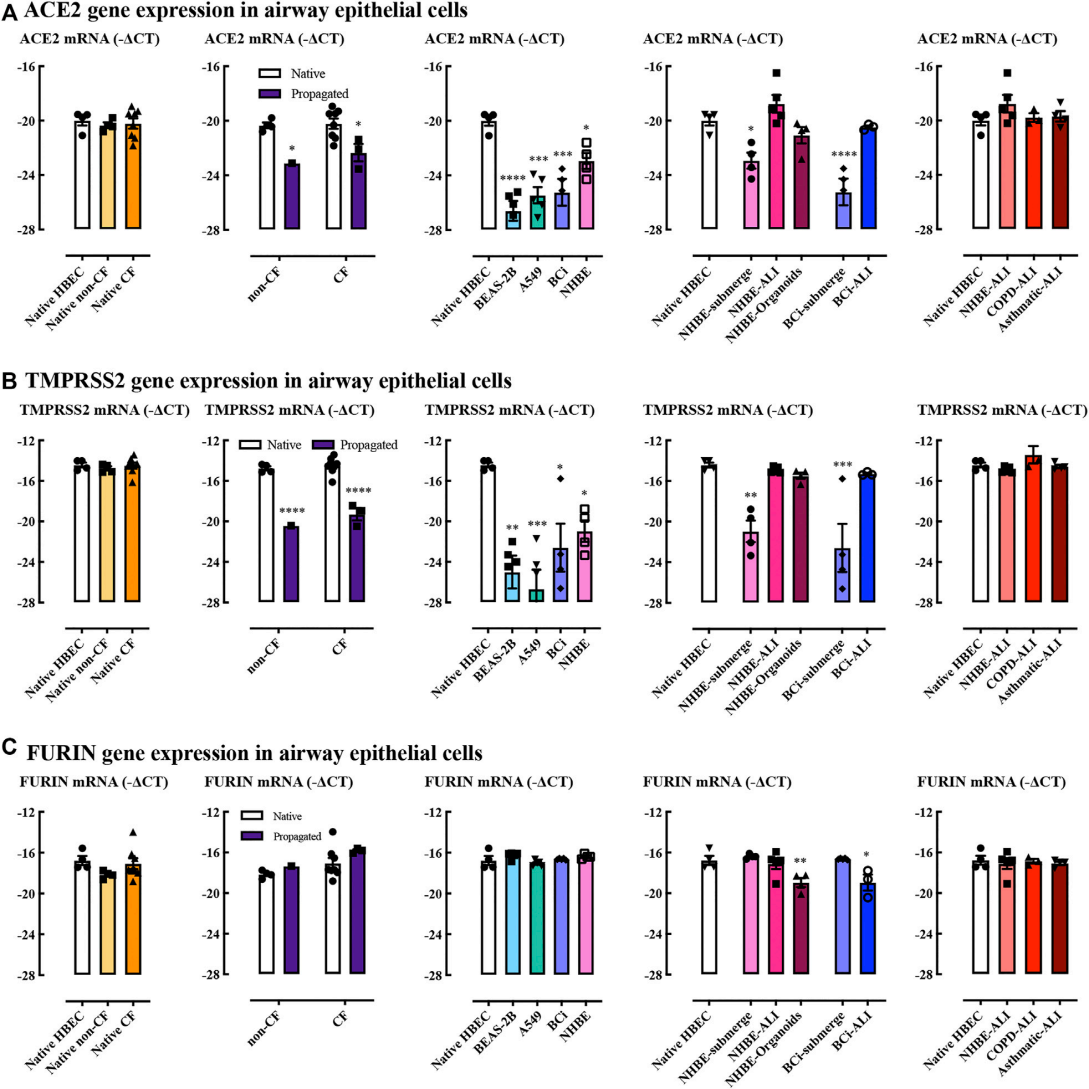
ACE2, TMPRSS2, and furin gene expression in airway epithelial cells. Quantitative real-time PCR (RT-qPCR) analysis of ACE2 **(A)**, TMPRSS2 **(B)**, and FURIN **(C)** genes in different cells models. Respectively, *n* = 4 for native adult HBEC, *n* = 4 for native infant non-CF, *n* = 8 for native infant CF (native cells), *n* = 1 propagated infant non-CF, *n* = 3 for propagated infant CF, *n* = 5 for BEAS-2B, *n* = 5 for A549, *n* = 5 for BCi, *n* = 4 for NHBE, *n* = 5 for NHBE-ALI, *n* = 4 for NHBE-organoids, *n* = 3 for BCi-ALI, *n* = 3 for COPD-ALI, *n* = 4 for asthmatic-ALI. Gene expression is expressed as −∆CT (Log_2_). Data are presented as mean and SEM. One-way ANOVA and Two-way ANOVA tests were used for analysis, *: *p* < 0.05, **: *p* < 0.01, ***: *p* < 0.001, ****: *p* < 0.0001.

ACE2 and TMPRSS2 genes were expressed to similar levels in native epithelial cells derived from adult and infant donors (Figures 2A,B). There was also no obvious difference between non-CF and CF infants. ACE2 and TMPRSS2 gene expression levels markedly declined upon subjecting primary cells to submerged cell culture conditions. BEAS-2B and A549 cells expressed significantly lower levels of ACE2 and TMPRSS2 than native cells. BCi and NHBE cultured at ALI showed restoration of ACE2 and TMPRSS2 expression levels compared to submerged culture. NHBE organoids showed a restoration of ACE2 and TMPRSS2 to near-native expression levels. Furin gene was expressed at similar levels in both adult and infant native cells (Figure 2C), and was not altered by propagation. Interestingly, the expression level of furin gene in submerged cultured cells was not reduced in contrast to the impact of submerged culture on to ACE2 and TMPRSS2 expression. However, NHBE organoids and BCi-derived ALI cultures showed decreased furin expression. In general, ACE2, TMPRSS2, and furin gene expression in ALI cultures derived from COPD and asthma patients did not appear to be different in this limited set of samples.

### ACE2 Is Highly Expressed in Ciliated and Basal Epithelial Cells

**FIGURE 3:**
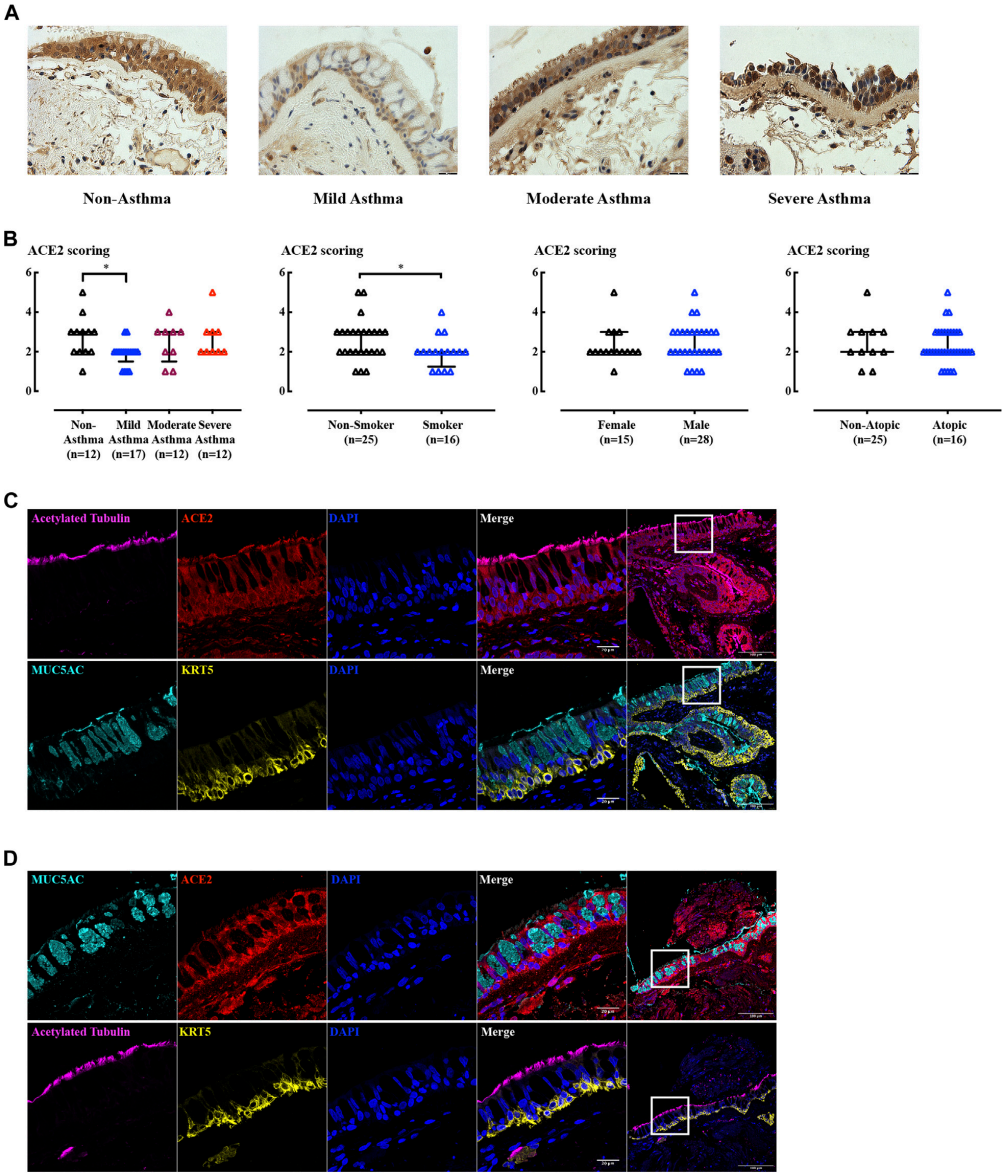
IHC and IF staining of ACE2 in human airway biopsies. **(A)** Representative ACE2 immunoreactivity in airway biopsies from subjects with increasing asthma severity. Scale bar = 180 µm. **(B)** Blinded assessments of ACE2 levels (on a scale of 0–5) and tested by non-parametric Mann-Whitney test. *: *p* < 0.05. **(C–D)** Immunofluorescence staining of acetylated tubulin (magenta), CC10 (green), MUC5AC (cyan), KRT5 (yellow), ACE2 (red), and nucleus (blue) in non-smoking, non-asthma subject **(C)**, and smoking, mild-asthma subject **(D)** paraffin sections. The left panel images were obtained using 63x objective and were taken from the highlighted area in the corresponding right hand side panel (20x objective).

ACE2 protein expression levels were measured in biopsies from non-asthmatic subjects, patients with mild, moderate, and severe (steroid-resistant) asthma (Figure 3A). Mild asthma subjects had slightly lower levels of ACE2 compared to non-asthma control subjects (Figure 3B). Re-analysis of the ACE2 expression excluding smokers did not change the pattern of ACE2 expression with a significant change only in the mild asthma group (data not shown). ACE2 expression was not influenced by sex or atopic status, nor usage of inhaled corticosteroids (ICS) or long acting β_2_-agonist (LABA) (data not shown). In contrast, ACE2 expression was lower in smokers compared to non-smokers. Interestingly, in BCi cell derived ALI cultures, ACE2 gene expression was not altered by budesonide (ICS), alone or in combination with formoterol (LABA), and dexamethasone (Supplementary Figure S2B). However, pilot data showed that dexamethasone tended to increase ACE2 gene expression when hydrocortisone was removed from the medium (Supplementary Figure S2C). This suggests that the medium composition has a potentially confounding effect when considering effects of synthetic ICS, as we have observed previously (Prodanovic et al., 2017).

Goblet cell and submucosal gland enlargement are the typical structural changes in COPD airway remodeling (Bergeron et al., 2009; Wadsworth et al., 2012). The reduced ACE2 in smokers may be explained by changes in cellularity as ACE2 expression in the airway epithelium proved to be cell type dependent in ALI and organoid cultures (see below). Sequential 2 µm biopsy sections from both non-smoking, non-asthma subjects (Figure 3C) and smoking, mild-asthma subjects (Figure 3D) stained with cell type markers showed that ACE2 was present in cells expressing ciliated and basal cell markers. Regions of goblet cell hyperplasia in smoking, mild asthma donor cells were devoid of ACE2 expression, potentially explaining the lower level of receptor expression in this group.

**FIGURE 4:**
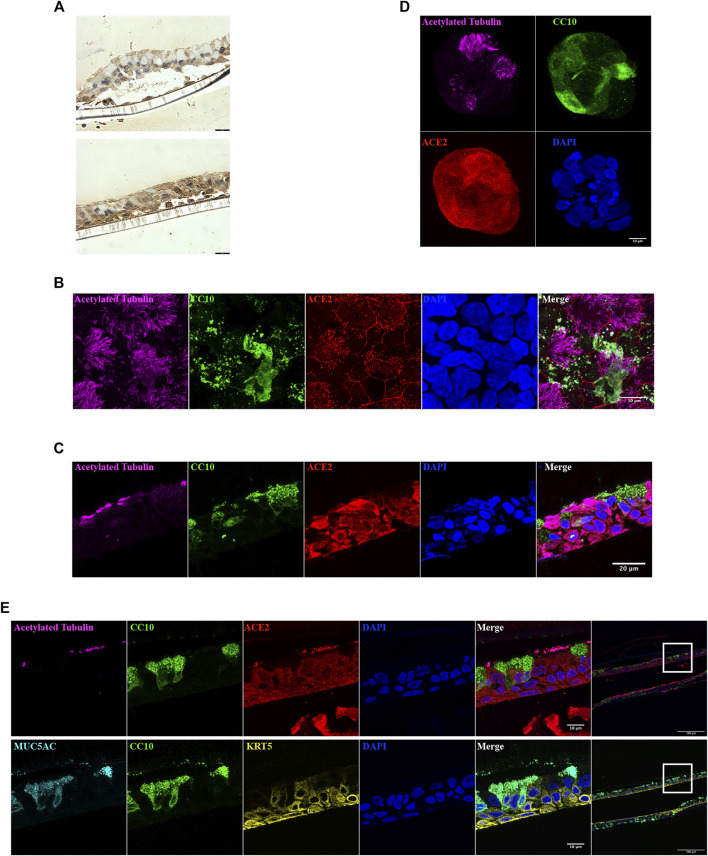
ACE2 localization in ALI and organoid cultures. **(A)** ACE2 immunoreactivity in NHBE cells derived ALI cultures. Images from same section. Scale bar = 180 µm. **(B–D)** Immunofluorescence staining of acetylated tubulin (magenta), CC10 (green), ACE2 (red), and nucleus (blue) in NHBE cells derived ALI **(B)**, BCi cells derived ALI **(C)** and NHBE cells derived organoid **(D)**. **(E)** Immunofluorescence staining of acetylated tubulin (magenta), CC10 (green), MUC5AC (cyan), KRT5 (yellow), ACE2 (red), and nucleus (blue) in 2 µm NHBE cell derived ALI paraffin sections. The left panel images were obtained using 63x objective and were taken from the highlighted area in the corresponding right hand side panel (20x objective).

In ALI sections, ACE2 was readily identified, but was differentially distributed within the cell populations present (Figure 4A). Whole mount staining images suggested that ACE2 is expressed in both NHBE cell derived ALI (Figure 4B) and BCi cell derived ALI (Figure 4C). ACE2 immunoreactivity appeared to be attenuated at the cell membrane. ACE2 was also expressed at the cell membrane in both inside-out (Figure 4D) and inside-in organoids (Supplementary Figure S1C). Surface expression of ACE2 is yet to be confirmed as this apparent cell boundary attenuation of IF signal might be a consequence of the projection of the confocal images. In 2 µm ALI sections, ACE2 was expressed in basal cells and ciliated cells, with high expression in the cilia (Figure 4E), but was undetectable in secretory, club and goblet cells. In airway organoids, ACE2 was localized in cells with cilia, but not in secretory cells expressing CC10 (Supplementary Video S5). Thus, the ACE2 distribution observed in airway biopsies was recapitulated in the ALI and airway organoids*.*

### SARS-CoV-2 can Infect NHBE and BCi ALI

**FIGURE 5:**
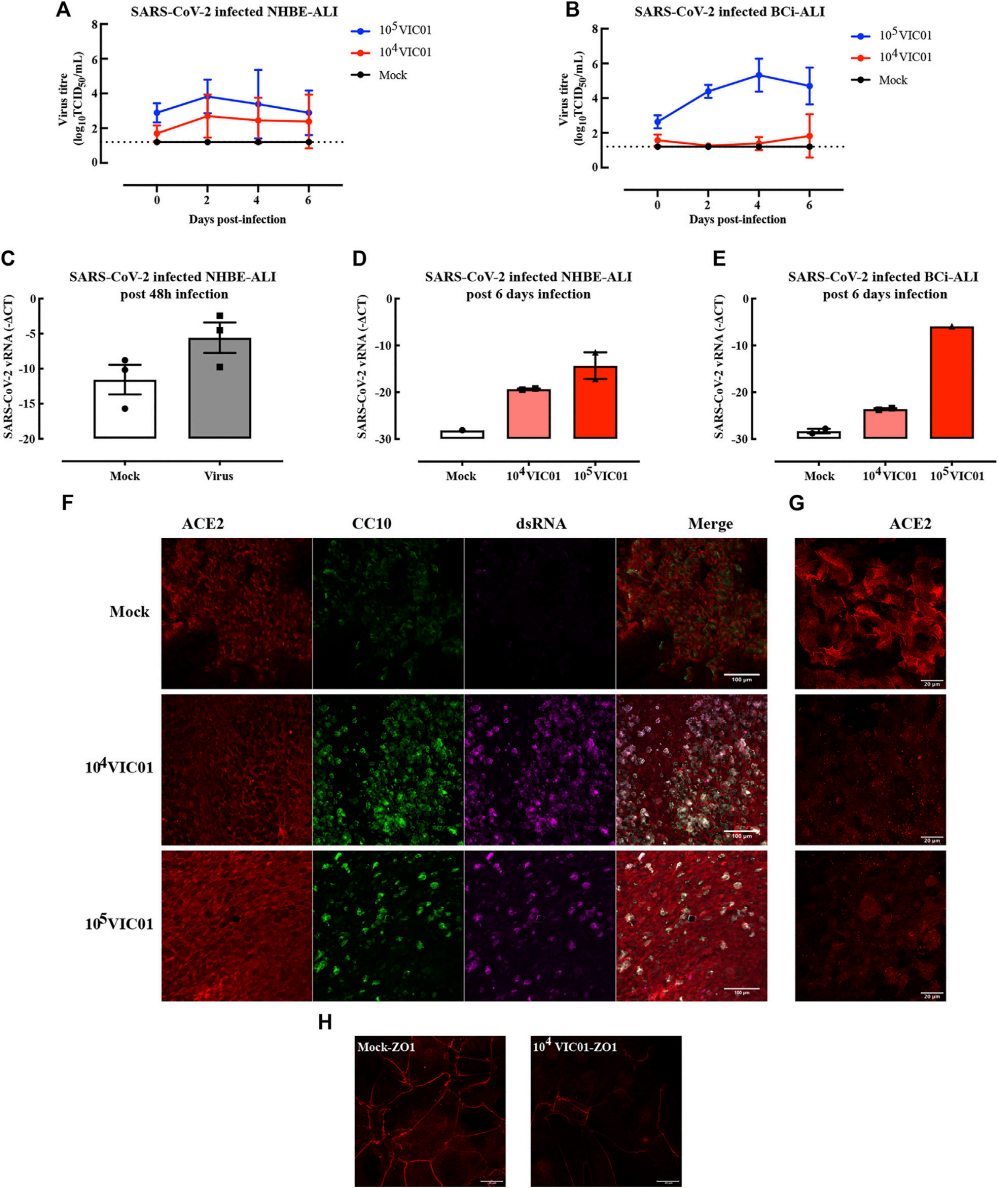
ALI cultures infected with SARS-CoV-2. ALI cultures were infected with SARS-CoV-2 for 1 h. NHBE cells from two independent donors were used in two separate infection experiments in which samples were collected at either post 48 h or post 6 days. Samples from SARS-CoV-2 infected BCi ALI cultures were analyzed at 6 days post infection. SARS-CoV-2 replication kinetics in NHBE cell derived ALI **(A)** and BCi cell derived ALI **(B)** were detected from apical washes. Gene expression of SARS-CoV-2 in NHBE-ALI was measured 48 h **(C)** and 6 days **(D)** post-infection, in BCi-ALI was measured 5 days **(E)** post-infection. Gene expression is expressed as −∆CT (Log_2_). Data are presented as mean and SEM for n = 1 independent culture with n = 3–4 technical repeats. SARS-CoV-2 infected BCi-ALI cultures were stained for ACE2 (red), CC10 (green), and dsRNA (magenta) at 6 days post-infection. Images were obtained using 20x objective **(F)** and 63x objective **(G)**. SARS-CoV-2 infected NHBE-ALI were stained with ZO-1 (red) at 6 days post-infection **(H)**.

To evaluate the infectivity of SARS-CoV-2, NHBE and BCi ALI cultures and submerged NHBE and BCi cells were inoculated with SARS-CoV-2. At the outset, airway organoids were deemed unsuitable, based on the physical/biosafety challenges of infecting organoids at biosafety level 3 and 4. Submerged NHBE and BCi cells were not susceptible to SARS-CoV-2 (data not shown). In contrast, both NHBE (Figure 5A) and BCi (Figure 5B) ALI cultures were infected with SARS-CoV-2, with peak virus shedding in the apical wash at 48–96 h post infection remaining detectable up to 6 days. Additionally, SARS-CoV-2 viral RNA (vRNA) was readily detectable in cells at 48 h (Figure 5C) and 5–6 days post infection (Figures 5D,E). However, there was no regulation of viral receptors nor induction of interferons by SARS-CoV-2 in either NHBE or BCi ALI at 48 h or 6 days post-infection (Supplementary Figure S3A–F). Although IL-6 is elevated in the serum of patients with severe COVID-19 (Cummings et al., 2020), there was no significant inflammatory cytokine/chemokine induction by SARS-CoV-2 in our *in vitro* models (Supplementary Figure S3G), whereas in separate experiments increased cytokine levels were induced in cells exposed to rhinovirus (Supplementary Figure S3I) or Poly I:C (Supplementary Figure S3H). Mean IL-6 concentrations remained at 2 pg/ml after SARS-CoV-2 infection, whereas IL-6 was increased 100-fold by Poly I:C.

To confirm viral tropism, SARS-CoV-2 (dsRNA, magenta) was co-stained with the viral receptor ACE2 (red) and club cell marker CC10 (green) (Figures 5F,G). At 5 days post infection, viral dsRNA was found in cells infected with at two different MOIs. Surprisingly, the virus seemed to be mostly colocalized with secretory cells, but not other cell phenotypes. ACE2 expression was diminished compared to mock-infected cells, especially at cell membranes (Figure 5G). Furthermore, the disruption of the tight junction protein ZO-1 (Figure 5H) suggested a loss of cell integrity following infection of SARS-CoV-2.

## Discussion

ALI organotypic cultures proved the most promising of airway cellular models for studies of SARS-CoV-2 infection. Both ALI and airway organoid organotypic culture re-expressed ACE2 and TMPRSS2, which were suppressed in primary epithelial cell cultures under submerged conditions which lack ciliated, secretory or goblet cell phenotypes. Moreover, benchmarking against ACE2 levels and distribution patterns in non-asthmatic and asthmatic biopsies, ALI and airway organoid culture clearly recapitulated the differential cell phenotype distribution of ACE2. The reproducibility and ease-of-handling of ALI prioritize it over airway organoid cultures for the study of infectious diseases, especially given biosafety constraints. SARS-CoV-2 infection of ALI did not provoke a detectable increase in cytokine levels; a finding that is perhaps to have been expected, given the frequency of asymptomatic infection (Oran and Topol, 2020). However, further studies are required using airway epithelial cultures from octogenarian or older donors and from patients with co-morbidities associated with risk of severe COVID-19.

Organoid culture technology has rapidly developed in the last 10 years; but airway organoid cultures are not as well established for lung/airway as for intestinal tissues (Sato et al., 2009). The variability of organoid morphology and function limits their reproducibility (Table 1). Developing “organoid-on-a-chip” could fulfill the requirements for production, control, and analysis of organoid microenvironment (Park et al., 2019). Nevertheless, the apical surface of ALI is predictably infectable without disruption of the pseudostratified epithelium. In contrast, organoids require inoculation by microinjection (Meyer-Berg et al., 2020) or mechanical disruption and re-embedding (Zhou et al., 2018).

**TABLE 1:** Comparison of ALI and airway organoid culture.

	ALI	Airway organoid
Mature protocol	+++	+
Easy handling	+++	+
Reproducibility	+++	+
Physiological mechanical environment	+	+++
*In vivo* architecture	++	+++
Patient specific	+++	+++
Time consuming	+++	+++
Confounding from media components	+++	+++

The collagen-coated transwell membrane in ALI cultures and the Matrigel in organoid cultures can be regarded as extracellular matrix (ECM) substrates, with the transwells providing a stiffer substrate. The cytoskeleton distribution induced by culture on different ECM may lead to different drug responses between ALI and organoid culture. The epithelial cells are uni-polar in ALI culture, but bi-polar in organoid culture. Bipolarity was unexpected and requires further investigation of the differential mechanical cues that may develop in Matrigel remodelled by the embedded epithelial cells. Everted organoids have been reported (Zhou et al., 2018), but the mechanism behind this morphogenesis is not known. The bipolarity of organoids certainly creates more unwanted and unphysiological heterogeneity that further compromises the utility of these structures.

The well-characterized immortalized basal cell line (BCi) retains differentiation potential over long-term culture (Walters et al., 2013) and is expandable to cell numbers that would support drug screening campaigns. The expression of ACE2 and TMPRSS2 in BCi-derived ALI reinforce its potential for studying SARS-CoV-2 and other infections.

ACE2 has been identified as the primary receptor of SARS-CoV-2, as it is for two other human coronaviruses, SARS-CoV and HcoV-NL63 (Kuhn et al., 2004; Hofmann et al., 2005; Hoffmann et al., 2020). The S1/S2 cleavage by furin occurs during biosynthesis of the S protein and formation of virus particle (Takeda, 2021). The S protein is further proteolytically cleaved by TMPRSS2 at the S2’ site to promote affinity for ACE2 and viral access to host cellular cytosol (Hoffmann et al., 2020). Thus, cleavage by furin and TMPRSS2 are critical for the infectivity of SARS-CoV-2. SARS-CoV-2 has acquired a variety of mutations with delta variant of major concern. The P681R mutation of the delta variant has been shown to enhance the cleavage of the S protein to S1/S2, resulting in increased infection (Liu et al., 2021). Targeting both the viral and host factors for SARS-CoV-2 entry may be a promising strategy for controlling viral infections. The TMPRSS2 inhibitor, camostat mesylate suppresses SARS-CoV-2 infection *in vitro* (Hoffmann et al., 2021), but has no beneficial effect in the clinical setting (Gunst et al., 2021). Combinations of TMPRSS2 and furin inhibitors may yet prove useful.

Interestingly, there was no apparent relationship of ACE2, TMPRSS2, or furin gene expression levels with asthma, cystic fibrosis or COPD status of native cells or ALI cultures. Consistent with our findings, ACE2, TMPRSS2, and furin mRNA expression levels in bronchial brushing samples and sputum cells have also been reported to be unaffected by asthma status and show no correlation with sex or age (Bradding et al., 2020; Peters et al., 2020). However, ACE2 gene expression level was decreased in lower airway epithelial cells and bronchial brushing samples from asthma patients (Jackson et al., 2020; Wark et al., 2021). In our investigation of ACE2 expression in airway epithelium, ACE2 levels did not consistently change with asthma severities. ACE2 levels only decreased in mild asthma. Compared to COPD patients who did not use ICS, ICS users are reported to have a reduced sputum expression of the ACE2 gene (Finney et al., 2021). However, regular ICS asthma therapy has recently been shown to be associated with an increased risk of hospitalization, ICU admission, or death (Bloom et al., 2021). We have not observed any influence of ICS on ACE2 expression *in vitro*. However, ALI differentiation media contain cortisol which may mask an effect of synthetic glucocorticoids (Prodanovic et al., 2017). The media for ALI and airway organoid cultures are highly bioactive and include growth factors, cortisol, potent TGF-β inhibitors, anti-oxidant reagents, and ROCK inhibitors that are necessary for cell proliferation and differentiation, but have the potential to compromise the predictive pharmacology of these cellular models (Prodanovic et al., 2017). Nevertheless, the relationship of asthma treatment to COVID-19 needs further mechanistic studies.

ACE2 expression was predominantly observed in ciliated epithelial cells consistent with single-cell transcriptomics data showing higher expression of ACE2 in ciliated cells compared with goblet cells, and with immunohistochemical staining in bronchial tissues (Hikmet et al., 2020; Lee et al., 2020; Gerayeli et al., 2021). TMPRSS2 and furin, like ACE2, are localized to ciliated cells (O'Sullivan et al., 2021). Thus, the higher the proportion of secretory cells, the lower the level of ACE2. It is inferred that the proportion of ciliated cells in the airway or in organotypic cellular models may result in different SARS-CoV-2 infectivity.

Early in the COVID-19 pandemic, severe respiratory illness was ascribed to a cytokine storm with blood IL-6 levels correlated with mortality (Hojyo et al., 2020; Zhou et al., 2020). Asymptomatic individuals exhibited lower levels of pro- and anti-inflammatory cytokines, suggesting a weaker innate response to SARS-CoV-2 infection (Long et al., 2020). However, plasma IL-6 levels in severe COVID-19 patients are much lower than those in cytokine release syndromes due to immunotherapy (Leisman et al., 2020). Interferon (IFN) beta (IFN-b) in combination with lopinavir and ritonavir was reported to reduce viral load and accelerate recovery (Hung et al., 2020). The rationale for IFN-1b supplementation is emphasized by the reported poor induction by SARS-CoV-2 of IFN-I and IFN-III (Blanco-Melo et al., 2020; Hadjadj et al., 2020; Vanderheiden et al., 2020), as also observed in our study. The peptide sequence used to generate ACE2 antibody is from the C-terminus and is part of the ectodomain that is subject to ADAM17 cleavage (Jia et al., 2009). The corollary of this observation is that the residual cellular product of cleaved ACE2 will not be detected by this antibody. These findings raise the possibility that SARS-CoV-2 infection triggers ACE2 cleavage, which is suggested to be protective of further infection (Taglauer et al., 2021), but carries the strong potential for host damage by affecting the ratio of Ang II and Angiotensin 1–7 (Wang et al., 2022). In addition, ACE2 shedding may explain discordance between single cell mRNA expression and IHC findings (Schweitzer et al., 2021).

To our knowledge, this is the first head-to-head comparison of ALI cultures and airway organoid cultures, providing a systematic examination of the utility of airway epithelial models with a view to achieve better predictive value in evaluating the clinical potential of therapeutics for COVID-19 and is benchmarked by reference studies in airway biopsies. Moreover, we showed that SARS-CoV-2 infected ALI culture lacks inflammatory cytokine/interferon response, an observation that is consistent with the overwhelmingly asymptomatic transmission of COVID-19. However, we acknowledged that there are some limitations of our work. Accumulating data suggests that asthma and other respiratory diseases are associated with severe COVID-19 symptoms. The lack of a linear relationship between ACE2 expression and asthma severity suggests that factors other than ACE2 expression may be important in attenuating the severity of COVID-19 in asthma. The asthma-associated factors influencing ACE2 expression *in situ* in asthmatics of differing severity may not persist *in vitro* due to the loss of the pro-inflammatory milieu of the asthmatic airway*.* Moreover, whilst the ALI culture *ex vivo* appears anatomically similar to the airway epithelium *in situ*, the functional interactions may yet be somewhat distorted by the non-physiological nature of the differentiation medium. In this study, we were measuring total expression of ACE2. Measurement of surface ACE2 expression may further identify its differential distribution in airway epithelium. Lastly, due to limitation in the scope of analytical work to be conducted in BSL3 or higher facility, the pathological changes of SARS-CoV-2 infected airway organotypic cultures are yet to be fully revealed.

Taken together, our comparison of the utility of different cell lines and cultures suggests that ALI cultures provide the greatest utility to model SARS-CoV-2 infection. SARS-CoV-2 infection of ALI culture showed pathological changes including morphogenic changes, but only limited inflammatory cytokine responses.

## Data Availability

The original contributions presented in the study are included in the article/Supplementary Material, further inquiries can be directed to the corresponding author.
