# Supplementary figures and images for: ACE2 Expression in Organotypic Human Airway Epithelial Cultures and Airway Biopsies
Source: Front Pharmacol. 2022 Mar 11;13:813087. doi: 10.3389/fphar.2022.813087 (PMC8963460; doi:10.3389/fphar.2022.813087)

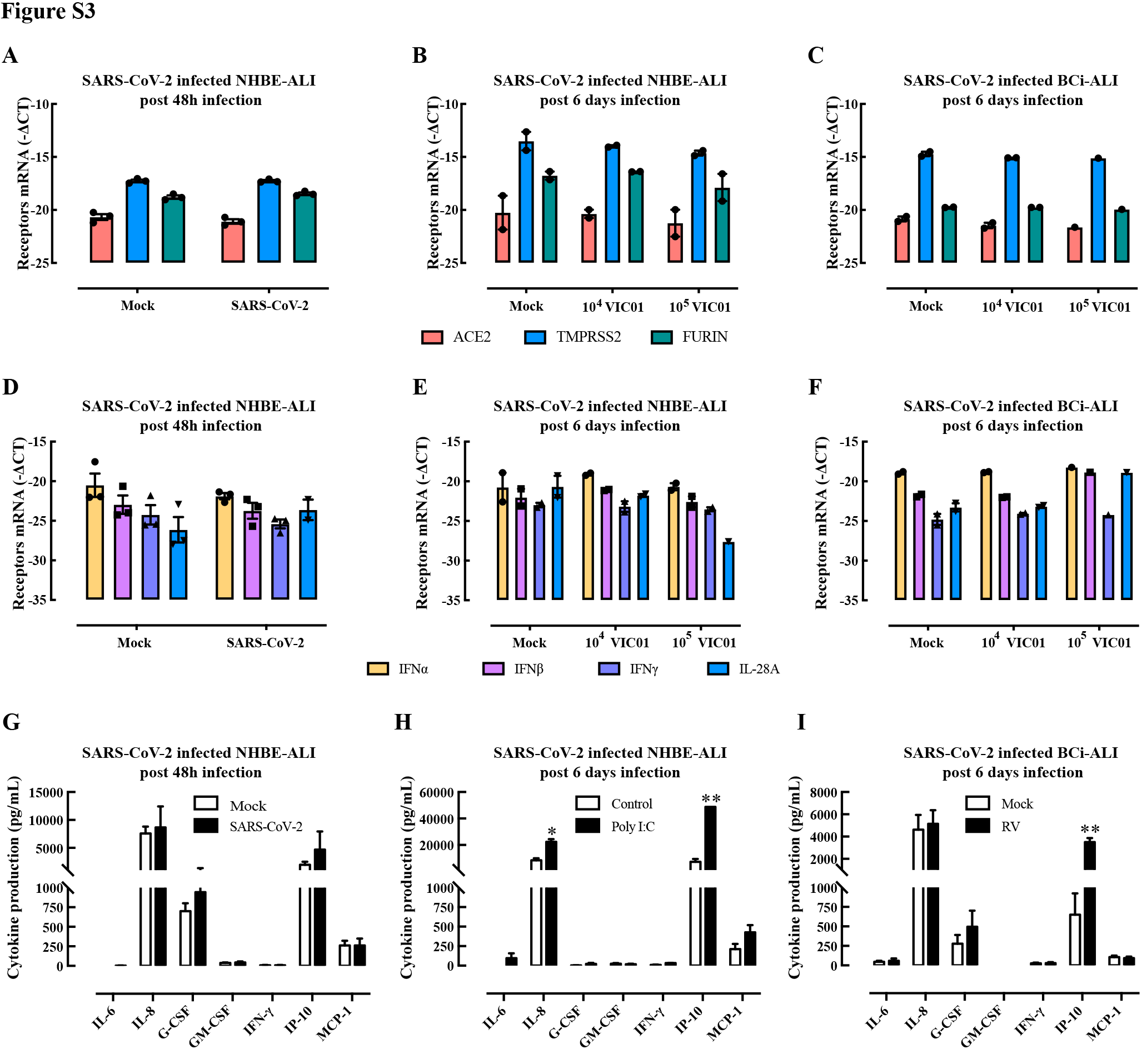

Supplement: Supplementary file 2 [file Image3.TIF]

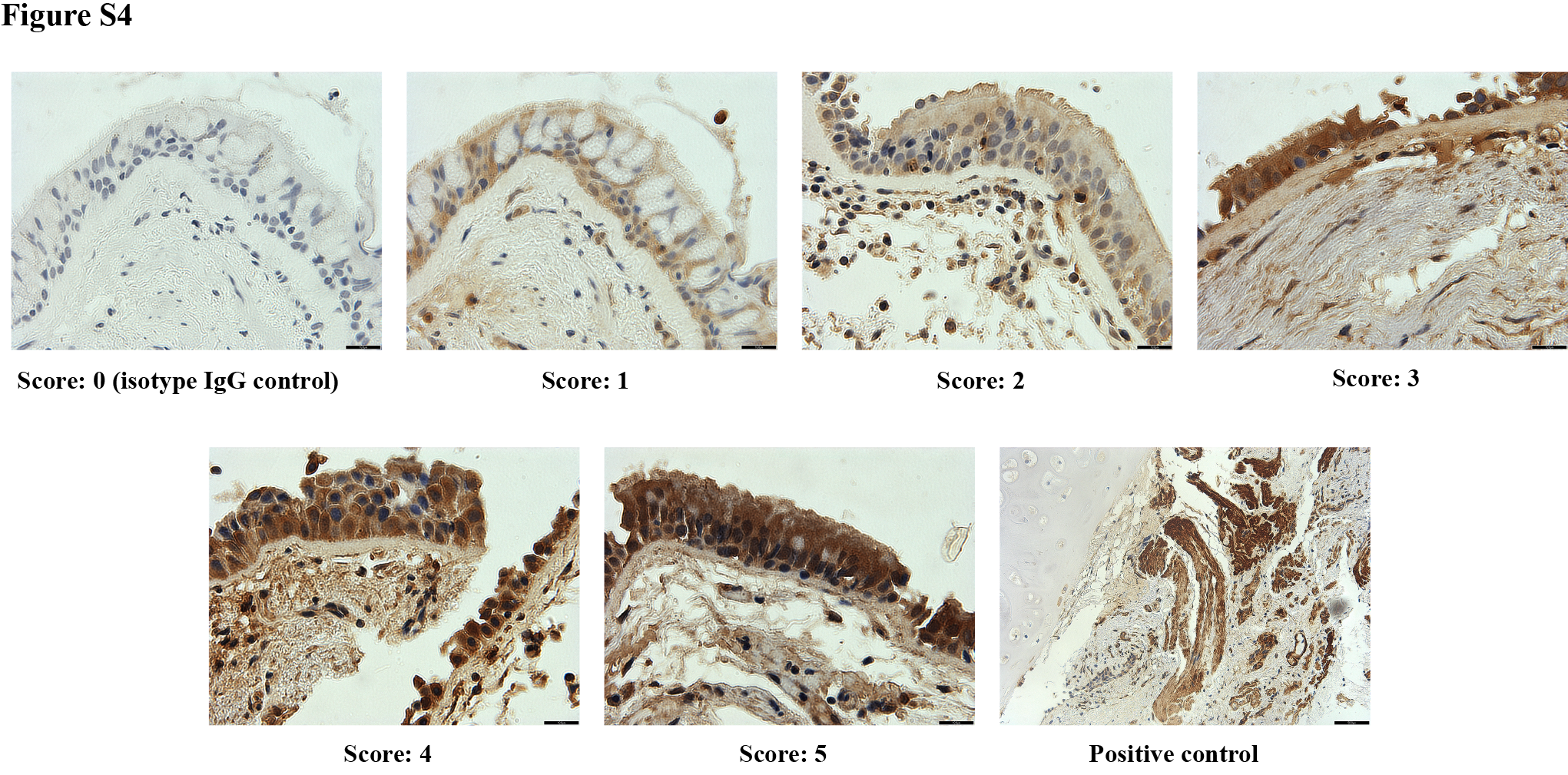

Supplement: Supplementary file 3 [file Image4.TIF]

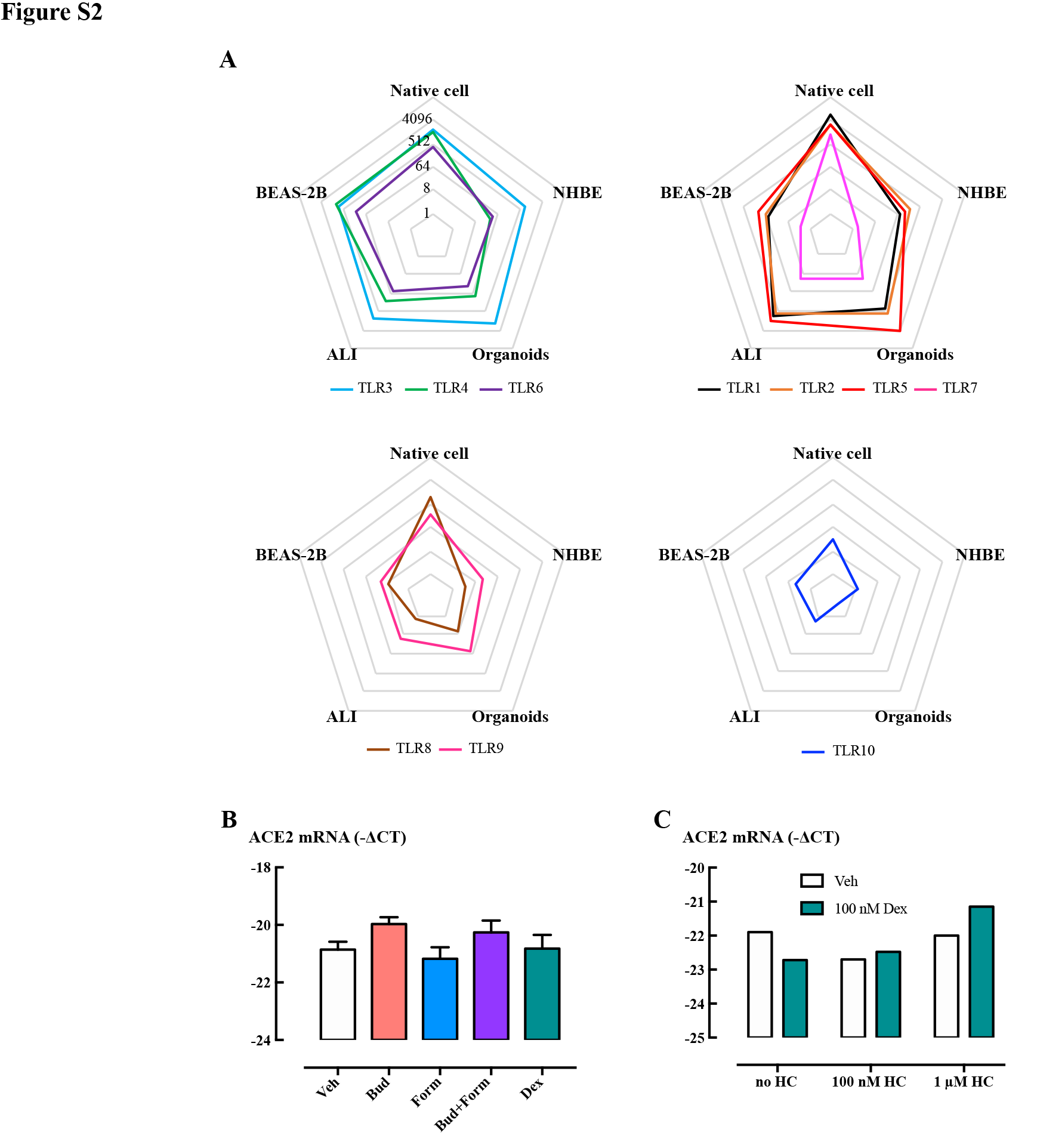

Supplement: Supplementary file 4 [file Image2.TIF]

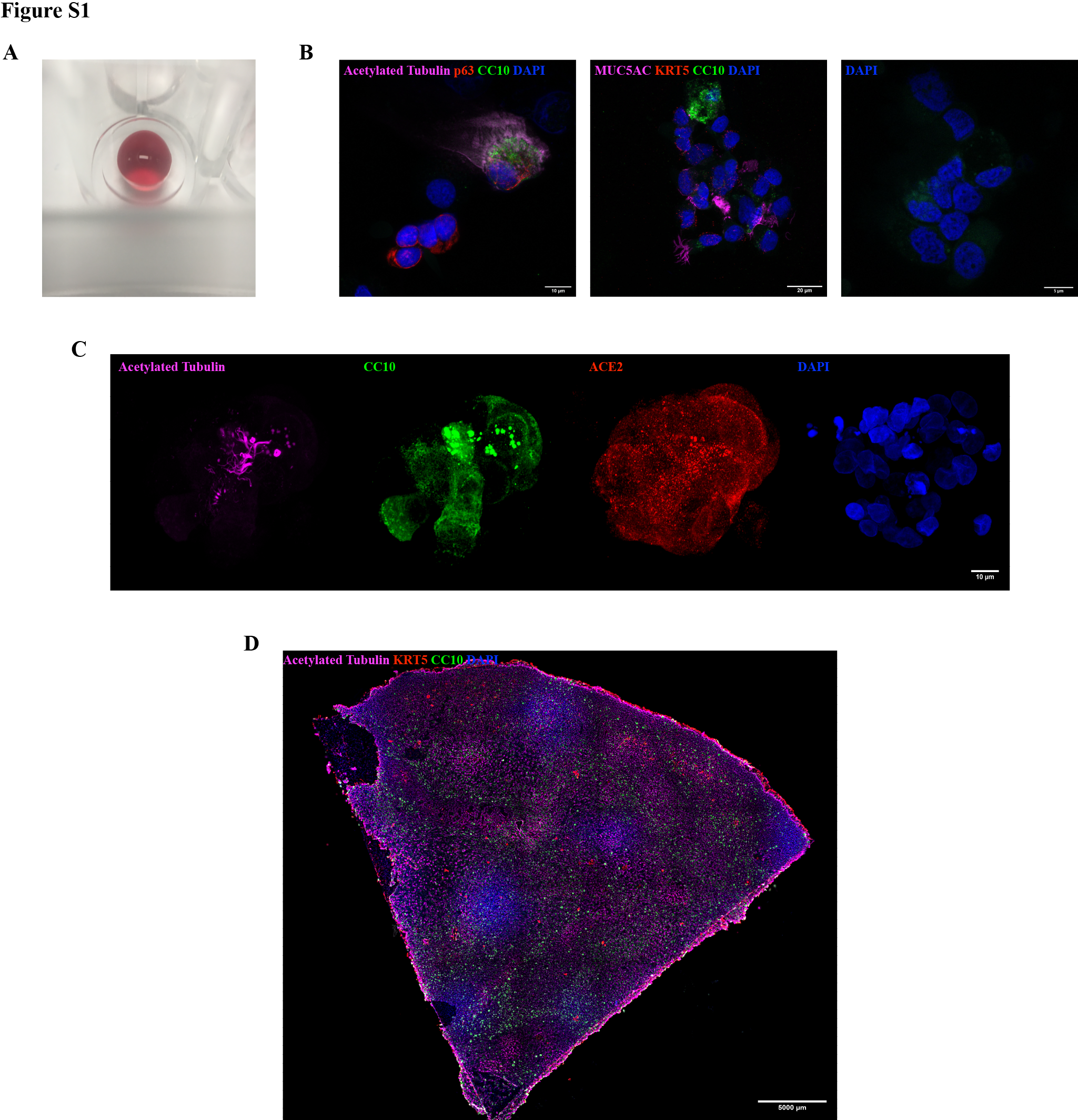

Supplement: Supplementary file 5 [file Image1.TIF]
