# Supplementary material for: ACE2 Expression in Organotypic Human Airway Epithelial Cultures and Airway Biopsies
Source: Front Pharmacol. 2022 Mar 11;13:813087. doi: 10.3389/fphar.2022.813087 (PMC8963460; doi:10.3389/fphar.2022.813087)
Supplement: Supplementary file 10 [file DataSheet1.docx]

Supplementary Material

ACE2 expression in organotypic airway epithelial cultures and airways biopsies

Qianyu Chen, Shenna Langenbach, Meina Li, Yuxiu C. Xia, Xumei Gao, Matthew J. Gartner, Elizabeth A. Pharo, Sinéad M. Williams, Shawn Todd, Nadeene Clarke, Sarath Ranganathan, Michelle L. Baker, Kanta Subbarao, Alastair G. Stewart

**Table S1.** Characteristics of donors of primary human bronchial epithelial brushing cell cultures

| **ID** | **CF**^*^**/Non-CF** | **Age** | **Sex** | **CFTR**^†^ **mutation 1** | **CFTR mutation 2** | **Modulator use** |
| --- | --- | --- | --- | --- | --- | --- |
| M1C130 | CF | 4.9 | Male | deltaF508 | G551D | Ivacaftor |
| M1C154 | CF | 3.2 | Male | deltaF508 | deltaF508 | No |
| M1C175 | CF | 1.0 | Female | c.1521_1523 (delCTT) | c.1521_1523 (delCTT) | No |
| M1C134 | CF | 5.0 | Male | delta F508 | delta F508 | No |
| M1C172 | CF | 2.0 | Male | delta F508 | delta F508 | No |
| M1C151 | CF | 4.0 | Male | delta F508 | delta F508 | No |
| M1C123 | CF | 6.0 | Female | delta F508 | delta F508 | Lumacaftor-ivacaftor |
| M1C126 | CF | 5.9 | Female | p.F508del | p.F508del | No |
| M1C129 | CF | 5.9 | Male | delta F508 | p.G551D | Yes |
| M1C158 | CF | 3.9 | Female | p.Arg1158Ter | p.Arg1158Ter | No |
| M1C176 | CF | 1.9 | Male | p.Phe508del | p.Phe508del | No |
| M1C184 | CF | 0.5 | Female | p.1507del | p.F508del | No |
| M1N050 | Non-CF | 8.4 | Female | N/A | N/A | N/A |
| M1N055 | Non-CF | 1.1 | Male | N/A | N/A | N/A |
| M1N056 | Non-CF | 5.9 | Female | N/A | N/A | N/A |
| M1N057 | Non-CF | 3.7 | Male | N/A | N/A | N/A |

^*^CF: cystic fibrosis; ^†^CFTR: cystic fibrosis transmembrane conductance regulator.

**Table S2.** Components of airway organoid medium

| **Media Component** | **Supplier** | **Final Concentration** |
| --- | --- | --- |
| R-Spondin 1 | 120-38, Peprotech, NJ, USA | 500 ng/mL |
| FGF 7 | 100-19, Peprotech | 25 ng/mL |
| FGF 10 | 100-26, Peprotech | 100 ng/mL |
| Noggin | 120-10C, Peprotech | 100 ng/mL |
| A83-01 | 2939, Tocris, UK | 500 nM |
| Y-27632 | Y0503, Sigma | 5 mM |
| SB202190 | S7067, Sigma | 500 nM |
| B27 supplement | 17504044, Life Technologies | 1x |
| N-Acetylcysteine | A9165, Sigma | 1.25 mM |
| Nicotinamide | N0636, Sigma | 5 mM |
| GlutaMax 100x | 35050061, Life Technologies | 1x |
| HEPES | H0887, Sigma | 10 mM |
| Penicillin / Streptomycin | P4458, Sigma | 100 U/mL / 100 mg/mL |
| Primocin | Ant-pm-1, Invivogen, CA, USA | 50 mg/mL |
| Advanced DMEM/F12 | 12634010, Life Technologies | 1x |

**Table S3.** Antibodies for immunohistochemistry and immunofluorescence

| **Name** | **Species** | **Dilution** | **Supplier** |
| --- | --- | --- | --- |
| Anti- Mucin 5AC [45M1] | Mouse monoclonal | 1:200 | ab3649, Abcam |
| Anti-ACE2 | Rabbit polyclonal | 1:200 (IF)/ 1:3000 (IHC) | ab15348, Abcam |
| Anti-Acetylated Tubulin | Mouse monoclonal | 1:4000 | T7451, Sigma |
| Anti-Cytokeratin 5 | Rabbit polyclonal | 1:1000 | ab53121, Abcam |
| E-Cadherin (24E10) | Rabbit monoclonal | 1:200 | 3195, Cell Signaling |
| Human Uteroglobin/SCGB1A1 | Rat monoclonal | 1:100 | MAB4218, R&D Systems |
| Anti-ZO-1 | Rabbit polyclonal | 1:100 | 61-7300, Invitrogen |
| Anti-dsRNA [J2] | Mouse monoclonal | 1:200 | Ab01299-2.0, Australian Biosearch |
| Alexa Fluor^®^ 488 Phalloidin |  | 1:50 | 8878S, Cell Signaling |
| IgG polyclonal isotype control | Rabbit polyclonal |  | ab37415, Abcam |
| Anti-Cytokeratin, pan antibody | Mouse monoclonal | 1:400 (IHC) | C2931, Sigma |
| Goat anti-Mouse IgG (H+L) Cross-Adsorbed Secondary Antibody, Alexa Fluor 647 |  | 1:500 | A21235, Invitrogen |
| Goat anti-Rabbit IgG (H+L) Cross-Adsorbed Secondary Antibody, Alexa Fluor 568 |  | 1:500 | A11011, Invitrogen |
| Goat anti-Rat IgG (H+L) Cross-Adsorbed Secondary Antibody, Alexa Fluor 488 |  | 1:500 | A11006, Invitrogen |

**Table S4.** Demographic data for subjects from the MESCA cohort from whom biopsies were obtained for immunohistochemistry

|  | **Non-Asthma** | **Mild Asthma ^*^** | **Moderate Asthma ^*^** | **Severe Asthma ^*^** |
| --- | --- | --- | --- | --- |
| Subject n (M/F) | 12 (7/5) | 17 (3/12 ^†^) | 9 (7/2) | 9 (2/7) |
| FEV_1_ %pred | 114 (98-141) | 102 (83-115) | 66 (50-73) | 64 (56-101) |
| Atopic (%) | 50 | 94 | 100 | 56 |
| Current smokers (%) | 0 | 53 | 56 | 50 ^‡^ |
| β­­_2_-adrenoceptor agonists (%) | 0 | 65 | 100 | N/A |
| Inhaled steroids (%) | 0 | 41 | 100 | 100 |
| Oral steroids (%) | 0 | 88 | 22 | 0 |

Data presented as median (interquartile range) or %. M: male; F: female; FEV­_1_: forced expiratory volume in one second; %pred: % predicted.

**^*^**: classified using Global Initiative for Asthma guidelines; ^†^: 2 unknowns; ^‡^: 5 unknowns.

**Table S5.** Human primer sequences for RT-qPCR

| **Gene product** | **Forward primer** | **Reverse primer** |
| --- | --- | --- |
| 18S | CGC CGC TAG AGG TGA AAT TC | TTG GCA AAT GCT TTC GCT C |
| ACE2 | GTT TGT AAC CCA GAT AAT CCA C | AAT GAT TTG CTC TTG CCA TC |
| DNAH1 | ACT AGT ACA AGA GGT CAT TAG G | CAC AGT ATT GTT GTA CAG GC |
| DNAH5 | CTT GAA AAA TGT TGT GAC CC | GTC ACC TTT ACA AAC AGA GAT G |
| FOXJ1 | GTG AAG CCT CCC TAC TC | AAT TCT GCC AGG TGG G |
| FURIN | AGA TGG GTT TAA TGA CTG GG | CAT AGA GTA CGA GGG TGA AC |
| IFNA | GTG AGG AAA TAC TTC CAA AGA ATC AC | TCT CAT GAT TTC TGC TCT GAC AA |
| IFNB | CAG CAA TTT TCA GTG TCA GAA GC | TCA TCC TGT CCT TGA GGC AGT |
| IFNG | AGC TCT GCA TCG TTT TGG GTT | GTT CCA TTA TCC GCT ACA TCT GAA |
| IL-28A | ACA TAG CCC AGT TCA AGT C | GAC TCT TCT AAG GCA TCT TTG |
| ITGA6 | CAA GGT CGT GAC ATG TGC TCA | TTC GTA TTA ACA TGC TGC CTT TTT T |
| KRT5 | AGT TTG TGA TGC TGA AGA AG | GTT AAT CTC ATC CAT CAG TGC |
| MUC5AC | GGA ACT GTG GGG ACA GCT CTT | GTC ACA TTC CTC AGC GAG GTC |
| MUC5B | TAC GTT CTG TCC AAG AAA TG | TAG ATG GAG TTG AGG AAC AC |
| SARSCOV2 | GCC TCT TCT CGT TCC TCA TCA C | AGC AGC ATC ACC GCC ATT G |
| Tektin 1 | ATT ACA GCT CTT GAA AAG GC | GGG CTA AAG TTT CCT TCA ATC |
| TFF3 | AGA ATG CAC CTT CTG AGG | AAA AGC TGA GAT GAA CAG TG |
| TLR1 | CCC TAC AAA AGG AAT CTG TAT C | TGC TAG TCA TTT TGG AAC AC |
| TLR2 | CTT TCA ACT GGT AGT TGT GG | GGA ATG GAG TTT AAA GAT CCT G |
| TLR3 | AGA TTC AAG GTA CAT CAT GC | CAA TTT ATG ACG AAA GGC AC |
| TLR4 | GAT TTA TCC AGG TGT GAA ATC C | TAT TAA GGT AGA GAG GTG GC |
| TLR5 | ATC TTT CAC ATG GGT TTG TC | TTC CCC CAG AAG GTT ATA TG |
| TLR6 | AGA GAT CTT GAA TTT GGA CTC | TGT CTT TGG TCA TGA TGT TG |
| TLR7 | AGA TAT AGG ATC ACT CCA TGC | CTT CCA AAA TGG AAT GTA GAG G |
| TLR8 | TGG AAA ACA TGT TCC TTC AG | TGC TTT TTC TCA TCA CAA GG |
| TLR9 | AAA TCC CTC ATA TCC CTG TC | TTG TAA TAA CAG TTG CCG TC |
| TLR10 | CAT CTG TAA GGG TTT TGA GC | CTT TCT TAG AGA CAT GTT GGA G |
| TMPRSS2 | CAG GTC ATA TTG AAC ATT CCA G | CTG AGT TCA AAG CCA TCT TG |

# Figures

Figure S1. (A) Airway organoid culture with silicone mask. (B) Detection of differentiated cells in native human bronchial brushing epithelial cells. Immunofluorescence staining for ciliated cells (acetylated tubulin, magenta), club cells (CC10, green), basal cells (p63 and KRT5, red), and nucleus (DAPI, blue). (C) Immunofluorescence staining of acetylated tubulin (magenta), CC10 (green), ACE2 (red), and nucleus (blue) in NHBE cells derived airway organoids with cilia towards inside. (D) Immunofluorescence staining of acetylated tubulin (magenta), CC10 (green), KRT5 (red), and nucleus (blue) in NHBE cell derived ALI. Confocal fluorescence images were acquired using Leica Confocal microscope with THUNDER Analysis (Leica).

**Figure S2.** (A) Comparison of TLRs gene expression in different airway epithelial cell models. The spider chart represents the quantitative real-time PCR (RT-qPCR) analysis of TLR 1-10 in native HBEC (n=4), BEAS-2B cells (n=5), submerged NHBE (n=4), NHBE-ALI (n=5), and NHBE-organoids (n=4). Gene expression is expressed as -∆CT (Log_2_). Interval equals to 8-fold difference. Data are presented as means. (B) BCi-ALI culture medium was changed to medium containing 100 nM hydrocortisone 24 h prior treatment of Budesonide (Bud) and/or formoterol (Form) and Dexamethasone (Dex). Gene expression of ACE2 is expressed as -∆CT (Log_2_) for n=3. (C) BCi-ALI culture medium was changed to medium containing 0, 100 nM, and 1 µM hydrocortisone 24 h prior Dexamethasone treatment. Gene expression of ACE2 is expressed as -∆CT (Log_2_).

**Figure S3.** ALI cultures were infected with SARS-CoV-2 for 1 h. NHBE cells from two independent donors were used in two separate infection experiments in which samples were collected at either post 48h or post 6 days. Samples from SARS-CoV-2 infected BCi ALI cultures were analyzed at 6 days post infection. Gene expression of ACE2, TMPRSS2, furin, and interferons in NHBE cell derived ALI was measured 48 h (A, D) and 6 days (B, E) post-infection, in BCi cell derived ALI was measured 5 days post-infection (C, F). Gene expression is expressed as -∆CT (Log_2_) for n=1 independent culture with n=3-4 technical repeats. Cytokines in supernatants were measured in ALI cultures 48 h post-infected with SARS-CoV-2 (G, n=3 technical repeats) and Rhinovirus (RV, I, n=4), and 24 h post-stimulated with 10µg/mL Poly I:C (H, n=3). Data are presented as mean and SEM. A paired-samples t-test was used for analysis, *: P<0.05, **: P<0.01.

**Figure S4.** Representative semi-quantitate IHC scoring. Scale bar = 180/50 µm.

# Videos

**Video S1.** Spheroid-like structure was generated 48h after native bronchial epithelial cells embedded in Matrigel^®.^

**Video S2.** Beating cilia was captured 14 days after native bronchial epithelial cells embedded in Matrigel^®.^

**Video S3-4.** Beating cilia in organoids cultured from two independent NHBE cultures.

**Video S5.** Immunofluorescence staining of acetylated tubulin (magenta), CC10 (green), ACE2 (red), and nucleus (blue) in NHBE cells derived organoid.
